# High Prevalence of Prediabetes and Associated Risk Factors in Urban Areas of Pontianak, Indonesia: A Cross-Sectional Study

**DOI:** 10.1155/2022/4851044

**Published:** 2022-12-10

**Authors:** Indah Budiastutik, Martha I. Kartasurya, Hertanto W. Subagio, Bagoes Widjanarko

**Affiliations:** ^1^Doctoral Program, Faculty of Public Health, Diponegoro University, Semarang 50275, Indonesia; ^2^Public Health Nutrition Department, Faculty of Public Health, Diponegoro University, Semarang 50275, Indonesia; ^3^Clinical Nutrition Department, Faculty of Medicine, Diponegoro University, Semarang 50275, Indonesia; ^4^Department of Health Promotion, Faculty of Public Health, Diponegoro University, Semarang 50275, Indonesia

## Abstract

Uncontrolled prediabetes can develop into Type 2 Diabetes mellitus (T2DM). The incidence of T2DM among adults in Pontianak, Indonesia was reported remarkably high. Therefore, this study aimed to investigate the risk factors for prediabetes in adults living in urban areas of Pontianak, Indonesia. A cross-sectional study was conducted in 5 subdistricts of Pontianak. A total of 506 adults underwent screening to obtain subjects with fasting blood glucose (FBS) of ≤124 mg/dL and aged >30 years. Blood pressure and body mass index (BMI) were measured. Interview using a structured questionnaire were performed to obtain data on predictor variables (age, sex, education, income, health insurance, tobacco use, history of hypertension, gout, high cholesterol level, frequency of exercise per week, and diabetic education). The prevalence of prediabetes among subjects was significantly high (76.4%). Subjects were predominantly above 40 years, female, had low income, low education level, and had health insurance. About a third of the subjects had a history of hypertension, gout, and high cholesterol level, respectively. The exercise frequency was mostly less than 3 times/week, and the BMI was mainly classified as overweight and obese. The result of spearman's rho correlation showed that age (*r* = 0.146; *p*=0.022) and BMI (*r* = 0.130; *p*=0.041) significantly correlated with prediabetes incidence. Moreover, the chi-square analysis demonstrated that health insurance ownership (OR = 4.473; 95% CI 1.824–10.972; *p* ≤ 0.001), history of hypertension (OR = 3.096; 95% CI 1.542–6.218; *p*=0.001), and history of gout (OR = 2.419; 95% CI 1.148–5.099; *p*=0.018), were associated with prediabetes incidence. For all these significant risk predictors except BMI, the significant associations were found only among female subjects after specific sex analysis. Moreover, multivariate logistic regression showed that health insurance ownerships (OR = 5.956; 95% CI 2.256–15.661; *p* ≤ 0.001) and history of hypertension (OR = 3.257; 95% CI 1.451–7.311; *p*=0.004), and systolic blood pressure (OR = 2.141; 95% CI 1.092–4.196; *p*=0.027) were the risk factors for prediabetes. It is concluded that the prevalence of prediabetes is probably high especially among urban people in Pontianak, Indonesia. Health insurance ownership and hypertension may have an important role in prediabetes management. The risk factors might be different between male and female.

## 1. Introduction

Prediabetes, also referred as impaired glucose tolerance, is a serious health problem which potentially develops to type 2 diabetes mellitus (T2DM). The blood glucose levels in prediabetes are higher than normal, but not high enough yet to be classified as diabetes [1]. The global prevalence of both prediabetes and diabetes are reported to increase rapidly [2]. The report showed that more than one-third of American adults had prediabetes, while over 84% were unaware of their condition, and about 5–10% progressed to diabetes. The prevalence of T2DM in Indonesia remains high, and the International Diabetes Federation has ranked Indonesia as the 7^th^ country with the highest number of cases, namely, about 10.7 million suffering from diabetes in 2019. Specifically, the prevalence of T2DM in West Kalimantan Province, Indonesia according to Indonesian Basic Health Research 2018 was 1.6% which was quite lower than the national prevalence (2.0%). Unfortunately, the data from Pontianak City Health Office, West Kalimantan in 2019 showed that the incidence of T2DM was 42% among people aged over 40 years [3]. Also, it was notably reported that the cumulative prevalence of prediabetes and T2DM sharply increased from 2007 (5.7%) to 2018 (10.9%) [4]. Furthermore, prediabetes is associated with an increased risk of all-cause mortality and cardiovascular diseases in the general population, and the risk gets higher in patients with atherosclerosis [5].

Several factors may generate prediabetes, while the prevalence increases with age and obesity [6]. According to the Centre for Disease Control and Prevention (CDC), the risk factors for prediabetes are overweight, age of 45 years or older, having a family history with T2DM, lack of physical activity (<3 times per week), gestational diabetes, and polycystic ovary syndrome [7]. In addition, a study found that there are other risk factors for prediabetes and T2DM including hypertension, low level of HDL-cholesterol, having a first-degree relative(s) with diabetes, previously having elevated blood glucose levels, and member of ethnic-minority communities [8]. Other risk factors have also been reported, such as smoking, low level of education and income, and being female [9].

Prediabetes is a condition that can be managed, which therefore could prevent from developing T2DM. The management can be done by screening the risk factors for prediabetes [10]. The screening involves both noninvasive and laboratory measures. The noninvasive method includes identifying age, sex, body mass index (BMI), blood pressure, family history, and lifestyle, while the laboratory measures include blood glucose tests [11]. Moreover, the criteria to define prediabetes and diabetes have been established by the American Diabetes Association where prediabetes is categorized by HbA1c test range of 5.7 to 6.4%, or fasting blood glucose (FBG) 100 to 125 mg/dL, or the oral glucose tolerance test 140 to 199 mg/dL [12].

Pontianak is located in Kalimantan Island which has a tropical climate and the fruit is supposed to be abundantly available. However, unlike other islands such as Sumatera and Java, the fruit availability is highly dependent on the season leading to irregular fruit consumption in Pontianak [13]. Moreover, fruit consumption is definitely correlated with the prevention of glucose intolerance [14]. In addition, the preferred foods are mostly high in energy, fat, and sugar, such as fried rice, noodle, fritters, and cake [15]. Therefore, this study aimed to investigate the risk factors for prediabetes among adults over 30 years in Pontianak where the diabetes incidence was very high recently.

## 2. Materials and Methods

### 2.1. Study Design

A community-based cross-sectional study was carried out in Pontianak, West Kalimantan Province, Indonesia. The design was used to determine the prevalence and risk factors of prediabetes. Data collection was conducted from February to April 2021 strictly following the standard COVID-19 prevention protocol provided by the Indonesian Ministry of Health. All protocols have been approved by the Health Research Ethics Committee in the Faculty of Public Health, Diponegoro University, Indonesia (Certificate of Approval No. 226/EA/KEPK-FKM/2020).

### 2.2. Target Population and Sampling Technique

**Figure 1:**
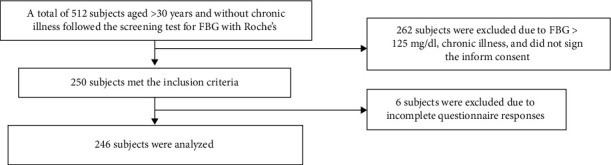
Flow diagram of subjects' recruitment.

The target population of this study was the inhabitants of all 5 subdistricts in Pontianak. Adult subjects were invited for screening tests, which were coordinated by head villages. From 512 participants in the screening tests, a total of 246 subjects met the inclusion criteria (aged >30 years, not pregnant, no history of chronic diseases, not under the treatment of oral antidiabetic, and willing to participate by signing the informed consent. Based on the power of 80% and 5% standard deviation, the calculated minimal sample size was 246 [16]. The flow diagram of subject recruitment can be seen in Figure 1.

### 2.3. Research Instrument and Measurement

The research instrument used in this study was a structured questionnaire which consisted of the questions about age, sex, education level, income, tobacco use, health insurance, history of some diseases including hypertension, gout, high cholesterol level, frequency of exercise per week, and participation to diabetes prevention education. The age of the subjects was divided into two categories (<40 years and ≥40 years). Based on the previous study, the age over 40 year increases the risk of developing diabetes [17]. Education level was grouped into low education level (primary and secondary school) and high education level (senior high school and higher education). Regional minimum wage/month was used to divide the income into low (<Rp. 2.515.000) and high (≥Rp. 2.515.000) [18]. Data of tobacco use, health insurance ownership, history of hypertension, gout, and high cholesterol level, participation to diabetes prevention education consisted of two categories (Yes or No). Exercise activity per week was made into two categories (≤3 times and >3 times for 30–50 min per exercise). Having exercise more than 3 times per week could improve the glycemic control and body composition of T2DM patient [19]. This study also measured the nutritional status of the subject using body mass index (BMI), which was calculated as weight in kilogram divided by the squared of height in meter. The BMI of the subjects were then stratified according to WHO classification as underweight (<18.5 kg/m^2^); normal (18.5–24.9 kg/m^2^); overweight (25–29.9 kg/m^2^), and obesity (≥30 kg/m^2^).

Hypertension status and FBG levels were measured at Prodia Clinical Laboratory, Pontianak (Accredited by National Accreditation Committee; ISO 15189:2012). Blood pressure was measured using a calibrated Omron M6 Comfort following the method of Bell and Williams [20]. Prior to measurement, subjects were asked to relax and remain seated for 25 minutes. The cuff was then placed on the left upper arm in a relaxed position. The subject was categorized as hypertension if the systolic blood pressure ≥140 mmHg and/or diastolic blood pressure ≥90 mmHg [21]. The FBG levels were measured by collecting 5 ml of a blood sample drawn from the antecubital vein. The blood samples were collected in the morning after the subject had fasted for approximately 8 hours. The automated glucose oxidase method was used to obtain FBG levels [22]. The subjects were categorized as having pre-diabetes if the FBG levels were between 100–125 mg/dL (according to American Diabetic Association criteria) [12]. FBS was used as the only parameter to diagnose pre-diabetes as FBS is an adequate, simple, safe procedure to diagnose prediabetes, suitable for a community-based study and also has a notable correlation with HbA1c [1, 23].

### 2.4. Data Analysis

Descriptive statistics were used to determine the frequency and percentage of all variables. The spearman's rho, chi-square tests, and multivariate logistic regression were performed to investigate the relationship between independent variables and prediabetes as the outcome in the present study. The association was considered as significant at *p* value of <0.05. In addition, the data were analysed using the IBM SPSS version 21 software with a Diponegoro University license (https://www.ibm.com/support/pages/downloading-ibm-spss-statistics-21).

## 3. Results

Of the 506 subjects who underwent screening, 246 subjects met the inclusion criteria. The subjects were mostly over 40 years (90.7%), female (80.5%), had a low income (60.6%) and education level (63.0%). More than half of the subjects were overweight or obese (65.5%). Predominantly, they had health insurance (71.5%). Tobacco use was relatively low among the subjects (36.6%). Most of the subjects had no history of hypertension (61.0%), gout (70.3%), and high cholesterol levels (65.9%). In addition, mostly the subjects had a frequency of exercise less than 3 times per week (79.3%). The subjects mainly did not participate in diabetic prevention education (61.0%). The measurements showed that 31.7% of the subjects had hypertension, where the prevalence of high systolic blood pressure (≥140 mmHg) and diastolic blood pressure (≥90 mmHg) were 48.0% and 38.6%, respectively. We notably found a high prevalence of prediabetes among the subjects, which reached 76.4% (188 subjects). The description of subjects' characteristics can be seen in Table 1.

The relationship between predictor variables and pre-diabetes status can be seen in Tables 2 and 3. The present study revealed that there was no association between sex, level of education, income, tobacco use, history of high cholesterol level, frequency of exercise, participation in diabetes prevention education, and prediabetes status. However, the Spearman's rho test showed a weak significant correlation between age (*r* = 0.146; *p* = 0.022), BMI (*r* = 0.130; *p* = 0.041) and prediabetes incidence (Table 2). Furthermore, according to chi-square test results, subjects with no health insurance ownership (OR = 4.473; 95% CI 1.824–10.972; *p* ≤ 0.001), history of hypertension (OR = 3.096; 95% CI 1.542–6.218; *p* = 0.001) and gout (OR = 2.419; 95% CI 1.148–5.099; *p* = 0.018) had a higher risk for pre-diabetes (Table 3). Due to wide differences in sex ratio, we conducted the separated analysis for males and females to investigate sex differences in the risk factor of pre-diabetes (Tables 2 and 3). Among male subjects, there was no significant predictor for prediabetes incidence. In contrast, among the female subjects, age (*r* = 0.184; *p* = 0.009), health insurance ownership (OR = 4.979; 95% CI 1.862–13.317; *p* = 0.001), history of hypertension (OR = 2.789; 95% 1.325–5.881; *p* = 0.010), and gout (OR = 2.576; 95% CI 1.122–5.912; *p* = 0.035) had significant associations with prediabetes status.

Table 4 shows the final logistic regression model of the risk factors for prediabetes for all subjects. All variables were introduced into a multivariate logistic regression. The results showed that health insurance ownership (OR = 5.956; 95% CI 2.256–15.661; *p* ≤ 0.01), history of hypertension (OR 3.257; 95% CI 1.451–7.311; *p*=0.004), and systolic blood pressure (OR = 2.141; 95% CI 1.092–4.196; *p*=0.025) were independently associated with the occurrence of pre-diabetes among the subjects.

## 4. Discussion

The present study found that the prevalence of prediabetes was remarkably high among the selected subjects in Pontianak. Two-third of the subjects had FBG >100 mg/dL. The prevalence was comparably with the prevalence of diabetes according to the data from the local health office. Indeed, a previous study in Uganda reported the increased rate of prediabetes (106%) coincided with the increase in obesity, central obesity, and diabetes prevalence [24]. This study is in line with our current study that found a high prevalence of obesity among the subjects. The significant increase in the prevalence of prediabetes has also been reported in England, the US, Iran, and Turkey [24–27].

Age, BMI, health insurance ownership, history of hypertension and gout, and systolic blood pressure were found to be significantly associated with prediabetes in the present study. Age has been reported to be a nonmodifiable risk factor for diabetes [28]. Our results showed that the significant association between age and prediabetes was observed only in females after sex-specific analysis. In women, age of ≥40 years is associated with having menopause, which is linked to body composition changes leading to the reduction of insulin sensitivity [29]. Moreover, this study revealed that BMI and prediabetes was significantly correlated with no sex-specific difference. Consistent to the present study, higher BMI has been reported as a strong risk factor of prediabetes [30]. The higher percentage of body fat produces larger amounts of free fatty acids, glycerol, and proinflammatory cytokines that participating in the development on insulin insensitivity [31].

From the multivariate model, subjects with no health insurance were at 5.9 times higher to have pre-diabetes. The results was in line with the previous study showing that uninsured people had significantly lower diabetes control than insured people [32]. Having no health insurance might indicate that the subjects did not pay much attention to their health status thus increasing the risk of diseases vulnerability [33]. Lack of health insurance ownership is commonly a limitation for obtaining routine and preventive therapy which are crucial for people with prediabetes where regular medical check-ups to monitor diseases progression is essentially important [34]. Besides providing medications, the health insurance could provide the basic information of health such as nutrition education which is the fundamental strategy to prevent prediabetes from developing T2DM [35]. In addition, people with health insurance have significant use of healthcare which indicates high awareness of health status [36, 37].

Hypertension has an important role in pre-diabetes incidence. Both from the results of chi-square analysis and multivariate logistic regression, a history of hypertension was significantly associated with prediabetes. In addition, the measured systolic blood pressure was found to be independently associated with prediabetes. The risk of prediabetes was 3.3 and 2.14 times higher in people with a history of hypertension and high systolic blood pressure, respectively. Hypertension is a degenerative disease that still be the major public health problem in Indonesia [38]. Moreover, hypertension is also a major risk factor for glucose intolerance and diabetic complication [39, 40]. High blood pressure is correlated with lower insulin sensitivity leading to impaired glucose uptake [41]. High insulin levels can increase sodium retention in renal tubules which can causatively induce hypertension [42]. This fact supports the statement of diabetes and hypertension are closely correlated and have a mutual interdependent effect [43]. Furthermore, our chi-square analysis revealed a history of gout might be the risk factor for prediabetes. Several studies reported people with gout have a higher risk of developing T2DM [44–46]. Higher uric acid levels have a strong correlation with higher adiposity and increased visceral fat deposition leading to insulin resistance [47] However, along with age and BMI, a history of gout did not significantly relate to prediabetes based on the multivariate analysis results. We propose susceptible multicollinearity between these independent variables. Age is a nonmodifiable risk factor for obesity, hypertension, and gout [48–51]. In addition, hypertension, obesity, and gout have been found to be correlated with each other [45, 52].

After sex-specific bivariate analysis, we found the significant associations between age, health insurance ownership, history of gout, and hypertension only among female subjects, but not in males. Some other factors including consumption of sugar-sweetened beverages, alcohol, intergenerational transmission of diabetes, sleep deprivation, and work stress may be more pronounced in males that is proposed to be the reasons of the findings [53]. In addition, a limited number of male subjects might also cause the insignificant associations.

There are some limitations and strengths in the present study. This study used a cross-sectional design in which exposures and outcomes variables were obtained in the same period. Therefore, the causality relationship could not be proven. The subjects were also not well distributed in age and sex which might cause no significant association for several variables. Also, menopausal status, family history of diabetes, history of gestational diabetes, food consumption pattern, caffeine intake, or any hormonal treatment were not considered. We also did not measure the insulin and adiponectin levels, while the insulin resistance is the most common factor present in prediabetic and adiponectin signaling plays an important role in both prediabetes and newly diagnosed diabetic [54]. However, the findings in the present study reported the recent update of a probably high prevalence of prediabetes among people living in a tropical urban area. The results also emphasize the role of health insurance and hypertension monitoring in T2DM prevention.

## 5. Conclusions

The present study found that the prevalence of pre-diabetes among adults over 30 years in urban areas of Pontianak, Indonesia, was remarkably high. Also, the study found a high prevalence of hypertension among the subjects. This study suggests that health insurance ownership and hypertension may play important roles in the incidence of prediabetes among the subjects, especially in females. This study implies that encouraging people of having insurance is essential for diabetic prevention.

## Figures and Tables

**Table 1:** Subjects' characteristics.

Characteristics	Frequency (*n* = 246)	Percent (%)
*Age (years)*		
≥40	223	90.7
<40	23	9.3
*Sex*		
Female	198	80.5
Male	48	19.5
*Nutritional status*		
Underweight (<18.5 kg/m^2^)	3	1.2
Normal (18.5–24.9 kg/m^2^)	82	33.3
Overweight (25–29.9 kg/m^2^)	119	48.3
Obese (≥30 kg/m^2^)	42	17.2
*Education levels*		
Low (primary, secondary)	155	63.0
High (senior high school, higher education)	91	37.0
*Family income*		
Low (<regional minimum wage)	97	39.4
High (≥regional minimum wage)	149	60.6
*Health insurance ownership*		
No	70	28.5
Yes	176	71.5
*Tobacco use*		
Yes	90	36.6
No	156	63.4
*History of hypertension*		
Yes	96	39.0
No	150	61.0
*History of gout*		
Yes	73	29.7
No	173	70.3
*History of high cholesterol level*		
Yes	84	34.1
No	162	65.9
*Frequency of exercise*		
≤3 times/week	195	79.3
>3 times/week	51	20.7
*Participation in diabetes prevention education*		
No	150	61.0
Yes	96	39.0
*Hypertension status*		
Hypertension (≥140/90)	78	31.7
Nonhypertension (<140/90)	168	68.3
*Systolic blood pressure*		
≥140 mmHg	118	48.0
<140 mmHg	128	52.0
*Diastolic blood pressure*		
≥90 mmHg	95	38.6
<90 mmHg	151	61.4
*Prediabetes status*		
Yes (FBG 101–125 mg/dL)	188	76.4
No (FBG <100 mg/dL)	58	23.6

**Table 2:** Spearman's rho correlation test's results between predictor variables and pre-diabetes status.

Independent variable	Male (*n* = 48)		Female (*n* = 198)		Total (*n* = 246)	
	Correlation coefficient	*p* value	Correlation coefficient	*p* value	Correlation coefficient	*p* value
Age	−0.005	0.972	0.184	0.009	0.146	0.022
Income	0.008	0.958	0.065	0.365	0.053	0.407
BMI	0.210	0.152	0.105	0.141	0.130	0.041
Systolic blood pressure	0.039	0.792	0.106	0.136	0.093	0.145
Diastolic blood pressure	−0.231	0.114	0.073	0.305	0.022	0.731

**Table 3:** Chi-square association between predictor variables and pre-diabetes status.

Independent variables	Male (*n* = 48)			Female (*n* = 198)			Total (*n* = 246)		
	Prediabetes		OR (95% CI)	*p* value	Prediabetes		OR (95% CI)	*p* value	Prediabetes		OR (95% CI)	*p* value
	Yes	No			Yes	No			Yes	No
*Sex*												
Female	—	—	—	—	—	—	—	—	150	48	1.21 (0.56–2.62)	0.618
Male	—	—	—	—	—	—	—	—	38	10	1	
*Education levels*												
Low (primary, secondary)	21	5	1.235 (0.306–4.983)	0.766	100	29	1.310 (0.670–2.563)	0.537	121	34	1.275 (0.43–1.43)	0.429
High (senior high school, higher education)	17	5	1		50	19	1		67	24	1	
*Health insurance ownership*												
No	9	1	2.793 (0.310–25.137)	0.343	55	5	4.979 (1.862–13.317)	0.001	64	6	4.473 (1.824–10.972)	≤0.001
Yes	29	9	1		95	43	1		124	52	1	
*Tobacco use*												
Yes	12	3	1.077 (0.237–4.902)	0.924	57	18	1.022 (0.522–1.998)	0.950	69	21	1.022 (0.554–1.884)	0.945
No	26	7	1		93	30	1		119	37	1	
*History of hypertension*												
Yes	16	1	6.545 (0.752–56.985)	0.059	68	11	2.789 (1.323–5.881)	0.010	84	12	3.096 (1.542–6.218)	0.001
No	22	9	1		82	37	1		104	46	1	
*History of gout*												
Yes	12	2	1.846 (0.339–10.043)	0.474	51	8	2.576 (1.122–5.912)	0.035	63	10	2.419 (1.148–5.099)	0.018
No	26	8	1		99	40	1		125	48	1	
*History of high cholesterol level*												
Yes	12	3	1.077 (0.237–4.902)	0924	56	13	1.604 (0.783–3.287)	0.261	68	16	1.488 (0.778–2.884)	0.228
No	26	7	1		94	35	1		120	42	1	
*Frequency of exercise*												
≤3 times/week	28	9	0.311 (0.035–2.776)	0.275	121	37	1.240 (0.565–2.721)	0.740	149	46	1.01 (0.48–2.07)	0.993
>3 times/week	10	1	1		29	11	1		39	12	1	
*Participation in diabetes prevention education*												
No	26	8	0.542 (0.100–2.947)	0.474	90	26	1.269 (0.659–2.444)	0.585	116	34	1.137 (0.624–2.071)	0.674
Yes	12	2	1		60	22	1		72	24	1	
*Hypertension status*												
Hypertension (≥140/90 mmHg)	14	3	6.545 (0.752–56.985)	0.059	48	13	1.267 (0.615–2.611)	0.644	62	16	0.77 (0.40–1.48)	0.440
Nonhypertension (<140/90 mmHg)	24	7	1		102	35	1		126	42	1	

^*∗*^1 = reference.

**Table 4:** Final multivariate logistic regression model of risk factors for prediabetes.

Predictors	OR	(95% CI)		*p* value
		Lower	Upper	
*Health insurance ownership*				
No	5.956	2.265	15.661	≤0.001
Yes	1			
*History of hypertension*				
Yes	3.257	1.451	7.311	0.004
No	1			
*Systolic blood pressure*				
≥140 mmHg	2.141	1.092	4.196	0.025
<140 mmHg	1			

^*∗*^1 = reference.

## Data Availability

The data sets used and/or analysed during the current study are available from the corresponding author on reasonable request.
